# Comparative Study Qualitative and Quantitative Techniques in the Study of Occlusion

**DOI:** 10.1155/2021/1163874

**Published:** 2021-09-23

**Authors:** Tanya Bozhkova, Nina Musurlieva, Diyan Slavchev

**Affiliations:** ^1^Department of Prosthetic Dentistry, Faculty of Dental Medicine, Medical University-Plovdiv, Bulgaria; ^2^Department of Social Medicine and Public Health, Faculty of Public Health, Medical University-Plovdiv, Bulgaria

## Abstract

**Introduction:**

The wide variety of methods for recording occlusal contacts and the contradictory data on the interpretation of the obtained markings provoked us to make a comparative laboratory study between different occlusal indicators.

**Purpose:**

Evaluation of a qualitative and quantitative method for registration of occlusal contacts in static occlusion in laboratory conditions.

**Materials and Methods:**

In completion of the objective, we designed an apparatus for registration of the occlusal contacts (AROC) in static occlusion which is used, corresponding to the MIP in clinical conditions. The occlusal indicators that were included in the study were articulating paper 100 *μ*, articulating foil 12 *μ*, and T-Scan Novus system with a sensor thickness of 100 *μ*. The collected primary statistical information was entered and processed with the statistical package SPSS Statistics 19.0, and the graphs were prepared using Microsoft Office 2019. We performed descriptive statistical analysis in this study. Comparisons were performed using one-way analysis of variance (ANOVA), Student's *t-*test, and Pearson coefficient method. For a significance level, *p* < 0.05 was chosen. *Result and Discussion*. With quality occlusal indicators, it is possible only to visually determine the size, number, and intensity of the marked contacts. After the statistical processing of the obtained data on the number of registered contacts, a significant difference is found in the number of contacts of certain teeth (18, 24, 25, 28, 38, 35, 34, 33, 44, 45, and 48) registered with articulating paper and articulating foil. The maximum force that is reported during the study with the T-Scan system is 93.72% and the forces in the right half of the dentition are 51.7% and in the left 48.9%. To visualize the location of the registered occlusal contacts with the system, it is intraoral to use a quality indicator and we recommend the use of articulating foil.

**Conclusion:**

Based on findings from the current in vitro simulation, we can conclude that the type of occlusal indicator influences the registration of contacts, and therefore, we propose as a method of choice to achieve a balanced occlusion in clinical practice to combine the use of one conventional and one quantitative method.

## 1. Introduction

The function of the masticatory apparatus is complete when the dentition is intact with contact between the individual teeth and proper occlusion with the antagonists. In this condition, the dentition is a single functional system, and the masticatory system is in functional equilibrium. The size, shape, and arrangement of the teeth are important for occlusion. Occlusion is a state of static contact between the teeth of the upper and lower jaws [1]. The healthy functioning of the masticatory apparatus is determined by the physiological restoration of the occlusion, as it is exposed to constant change [2].

Different methods are used in the study of occlusion—qualitative and quantitative [3–6]. The accuracy of the applied methods is important for the achievement of occlusal harmonic relations [7]. In dental practice, quality indicators are the most often used due to their lower cost and ease of use. Articulating paper (AP) is the most commonly used occlusal indicator [8]. According to Sharma et al. and Reiber et al., articulating foil (AF) is the thinnest occlusal indicator and more accurately registers occlusal contacts between teeth compared to articulating paper and articulating silk [9, 10].

### 1.1. Interpretation of the Strength of Occlusal Contacts according to the Size and Intensity of Their Staining

Despite numerous studies, dentists still consider the size of occlusal contact markings as an indicator of the strength of the load. The occlusal force can be determined by the size and intensity of the staining obtained on the occlusal surface of the teeth according to some authors [11–13]. Harper and Okeson argue that large and dark markers reflect a higher load, while small and light markers show a lower load [14, 15]. According to other authors, the intensity of the marking is an inaccurate criterion for assessing the strength of occlusal contacts [16–18]. In their study, Reddy et al. demonstrated that there was no correlation between the marking area and the applied occlusal load [19].

In practice, most dentists correct large dark markings obtained with articulating paper and ignore small dotted markings. The opposite is true: the areas with the highest load are marked as small points [20]. The size and intensity of the markings can vary, and the visual determination of occlusal forces is an inaccurate method [21]. Saad et al. and Carossa et al. have shown that a thicker occlusal indicator registers larger markings compared to a thinner one [22, 23]. With quality indicators, it is possible to determine only the location and number of occlusal contacts. Their disadvantage is that they cannot determine the sequence and strength of contacts and their interpretation is subjective [24–26]. It has been proven that with increasing the thickness of the occlusal indicator the number decreases and the area of the registered contacts increases [23, 25, 27].

### 1.2. Interpretation of the Strength of Occlusal Contacts according to Quantitative Data

The sequence, time, and strength of occlusal contacts can be determined by quantitative methods for the registration of occlusal-articulating relations [28, 29]. In 1984, Manes created the first computerized system, which underwent many changes, while in 2015 T-Scan Novus was created with software version 9.1. [30]. The T-Scan system is a reliable and proven method for occlusion registration [31–33].

The wide variety of methods for recording occlusal contacts and the existing contradictory literature data for the interpretation of the obtained markings provoked us to make a comparative laboratory study between different occlusal indicators. In conducting an in vivo study, we set ourselves the goal of comparing the obtained occlusal contacts with different methods and to assess the possibilities of occlusal indicators.

## 2. Purpose

The purpose is the evaluation of qualitative and quantitative methods in the registration of occlusal contacts in a static position in laboratory conditions.

## 3. Materials and Methods

**Figure 1:**
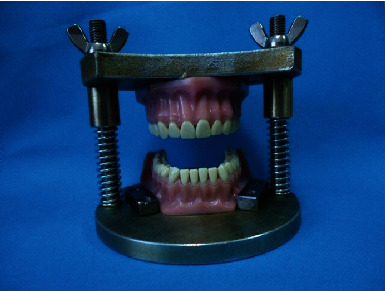
Apparatus for registration of the occlusal contacts.

To fulfill the set goal, a self-constructed apparatus for registration of the occlusal contacts in static occlusion (AROC) is used, corresponding to the MIP in clinical conditions. Its design reproduces strictly vertical movements of opening and closing. Standard phantom models of the lower and upper jaw with preserved tooth rows Frasaco A-3Z (Frasaco GmbH) are attached directly to the AROC device. The models are in class I angle occlusion. The device is designed so that the models are maximally stabilized and cannot be displaced when closed in the MIP. In this way, equal conditions are ensured in the registration of occlusal contacts. The device is designed to be able to withstand loads significantly exceeding the force used. The strength of the structure is guaranteed by the use of chromium-nickel (Cr-Ni) steel material and the solid thickness of the upper and lower plate (Figure 1) [34].

**Figure 2:**
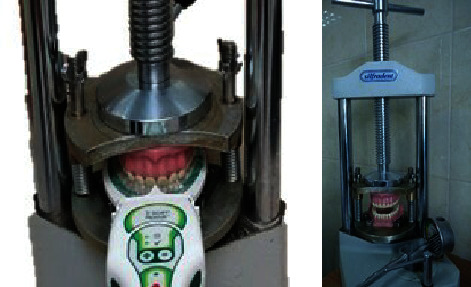
AROC device with the dental hydraulic press.

The occlusal contacts were registered in the MIP as the models were loaded with a force of 120 kg through a dental hydraulic press, model Silfradent. The force that develops during normal physiological chewing activity is called masticatory muscle strength (Pm). Its average value is about 30% of the absolute muscle strength (3822 N or 390 kg), approximately 1080-1180 N (110-120 kg) [34]. The occlusal indicators that were included in the study were articulating paper 100 *μ* (Bausch PROGRESS® 100, Dr. Jean Bausch GmbH & Co. KG, Koln, Germany), articulating foil 12 *μ* (Bausch Arti-Fol®, Dr. Jean Bausch GmbH & Co. KG, Koln, Germany), and the T-Scan Novus system (Tekscan, Inc., S. Boston, MA, USA) with a sensor thickness of 100 *μ* (Figure 2).

With each of the occlusal indicators, contacts were registered on the occlusal surfaces of the teeth, making 20 repetitions and applying the same force. Articulating paper strips and articulating foil strips were replaced every 3 repetitions to eliminate as much as possible the possibility of not registering markings due to tearing or reduction of the impregnating substance. The occlusal surfaces were cleaned after each contact marking. All T-Scan occlusion recordings were performed with a single sensor as it was not damaged. The location, number, and size of the occlusal contacts obtained with articulating paper and articulating foil were recorded with a digital camera Sony, under the same conditions.

The results obtained regarding the number of markings were plotted in tables for each occlusal indicator. The collected primary statistical information was entered and processed with the statistical package SPSS Statistics 19.0, and the graphs were prepared using Microsoft Office 2019. We performed descriptive statistical analysis in this study. Comparisons were performed using one-way analysis of variance (ANOVA), Student's *t*-test, and Pearson coefficient method. For a significance level, *p* < 0.05 was chosen.

The registered occlusal contacts with T-Scan Novus were recorded as a video and visualized as three- and two-dimensional images using the v 9.1 software. When reviewing the occlusion video, none were excluded from the sample, as they were all successful.

## 4. Results

**Figure 3:**
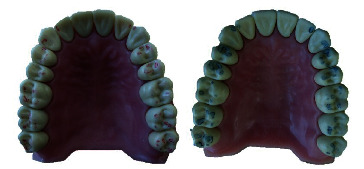
Registered occlusal contacts with articulating foil 12 *μ* and articulating paper 100 *μ*.

With quality occlusal indicators, only the visual determination of the location, size, number, and intensity of the marked contacts is possible (Figure 3).

The results obtained after statistical processing of the data on the number of registered contacts by qualitative methods (AF and AP) are presented in Table 1.

Table 1 presents the results of the registration of occlusal contacts with AF and AP. The value is assigned to each tooth accordingly as mean, std. deviation, std. error, minimum, and maximum number occlusal contacts.

After the statistical processing of the obtained data on the number of registered contacts, a significant difference is found in the number of contacts of certain teeth registered with articulating paper and articulating foil. The results are presented in Table 2, with a significance index of *p* < 0.05. Teeth with a statistically significant difference are stained.

A statistically significant difference in the number of contacts was found in the area of all third molars, in the area of the left upper and lower premolars, in the area of teeth 33, 44, and 45, respectively.

**Figure 4:**
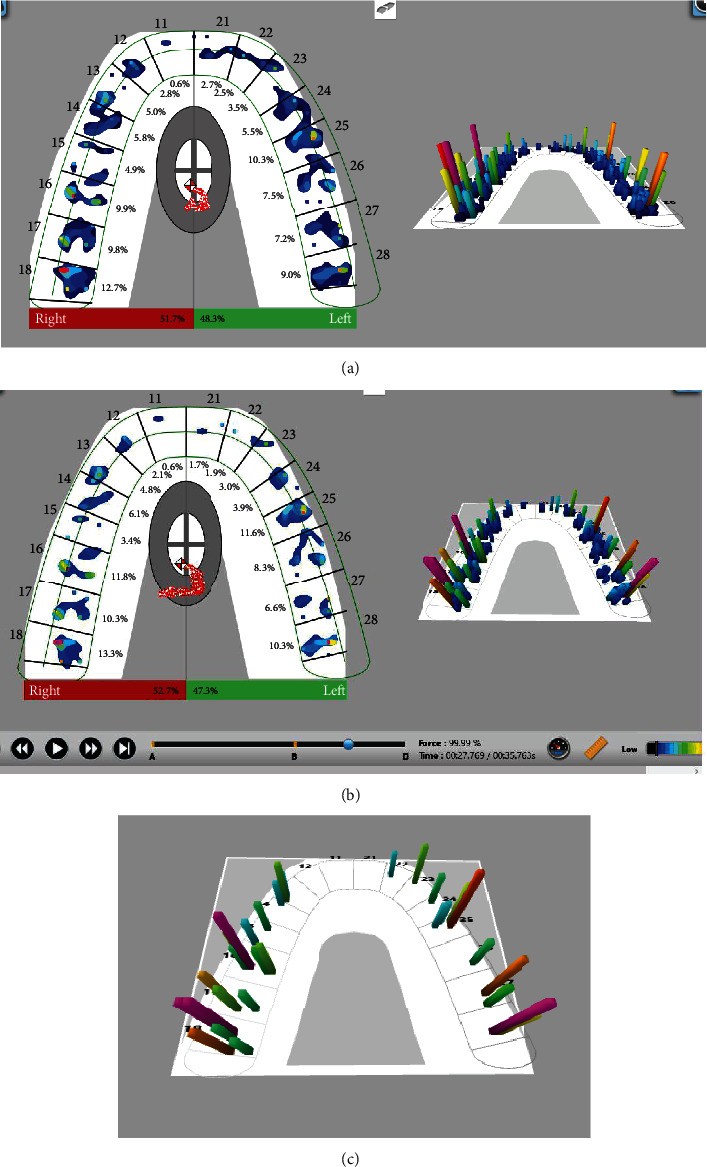
(a) Occlusal contacts in maximum bite forces. (b) Occlusal contacts without sensor compression. (c) Tooth contacts with high forces.

The sequence of contacts and their strength is evaluated using the T-Scan Novus system. Examination of all received recordings (force movie) shows that the distribution of occlusal contacts is the same for each one (Figure 4).

Examination of the video determined that the first contact occurred in the area of tooth 18, at the same time as teeth 17 and 27, and then tooth 25 came into contact. The maximum force reported during the examination was 93.72% and, respectively, the forces in the right half of the dentition are 51.7% and in the left 48.9%. COF is at the center of forces target all the time. The registered contacts are displayed on the upper dentition as two-dimensional and three-dimensional images.

The strength of the contacts is illustrated by color-coding from blue to red. Dark blue indicates compression of the sensor, which is not real contact between the teeth and usually appears near or around places of higher strength, coded with another color (green/yellow/red). By correcting the force legend, it is possible to eliminate the compression of the sensor, which makes it easier to analyze the actual points of contact (Figures 4(b) and 4(c)).

**Figure 5:**
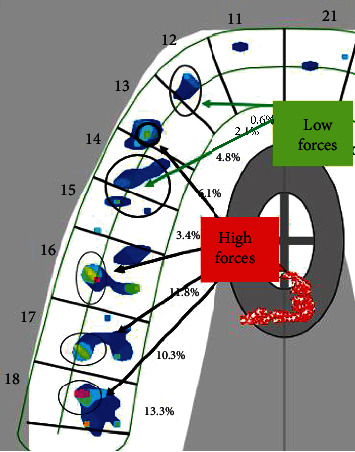
Occlusal contacts with the T-Scan system.

**Figure 6:**
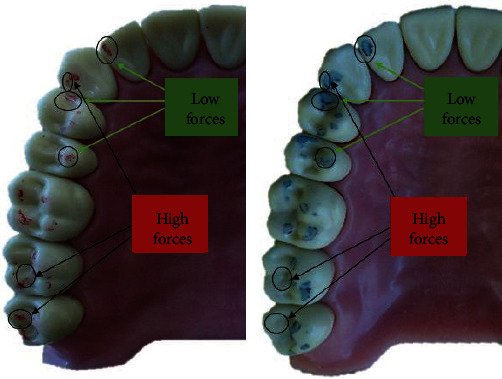
Occlusal contacts with articulating foil and articulating paper.

Visually defined as strong and weak contacts according to size and intensity are compared with contacts registered through the T-Scan system (Figures 5 and 6).

## 5. Discussion

A strong debate is continued about the strength of occlusal contacts based on their size and color.

Only the location of the contacts can be indicated by quality occlusal contacts. When comparing the results obtained by us with AP and AF, it is found that increasing the thickness of the indicator decreases the number of registered occlusal contacts. Our results coincide with those obtained by Millstein [18].

There is still a myth that the size and intensity of the staining of the marking can determine the occlusal force. This is an issue that continues to be discussed in several studies [11, 16, 19].

As can be seen from the markings obtained on the masticatory surfaces of the teeth, the occlusal contacts registered with articulating paper are larger than those obtained with articulating foil.

Our research confirms the statement that larger contacts are registered with thicker articulating paper [14, 23].

The variety in the number of contacts in the individual repetitions leads us to think that the quality indicators do not always manage to accurately register the actual contacts. Another study also demonstrated that the evaluation of occlusal contacts recorded with articulating paper was inaccurate and subjective, as positive false markings may be registered or not all occlusal contacts may be registered [17, 19, 26].

Many factors such as neuromuscular performance, saliva quality, quantity, tooth morphology, tooth mobility, occlusal surface type, roughness, and type of stain of the indicator material can affect the results of the occlusal analysis [35]. In 2002, a study was conducted to assess the sensitivity and reliability of articulating papers, foils, silk ribbons, and T-Scan systems on articulated models [7]. We find differences in the number of registered occlusal contacts in a device that reproduces only vertical movements. According to the classification of articulators according to “The Glossary of Prosthodontic Terms,” the devices are arranged in four classes. Class I are simple mechanical devices. They register a static position between the upper and lower jaws and allow only vertical movements [36]. With the classification formulated in this way, we can accept the apparatus we offer (AROC), for a mechanical device, an articulator of the first class.

Limitations to the current study: before the construction of our device AROC, a thorough study was conducted on the existence of such devices in our available literature (PubMed, Google Scholar, and other references). We aimed to reproduce strictly vertical movements to avoid unwanted formants, registered by the longer contact between the occlusal surfaces, after slipping on the main tubercles. When a TMJ-like element is introduced, the loading mechanism changes. There is a real risk of prolonged contact time between the occluding tooth surfaces, the formation of nonexistent contact relationships, which makes the logic of the study meaningless.

In trying to use articulators for the present study, we encountered problems of a structural nature. None of our available articulators has any technical documentation to guarantee the ability of the device to carry a load of 1080-1180 N (average value of the chewing force used in a normal chewing act (Black, 1895)).

The other problems we encountered are as follows:

1. They concerned the construction of the apparatus, which did not allow it to be carried out directly loaded with the used hydraulic press
2. The presence of an intermediary to fix the model may harm the study being conducted. The large layer of gypsum is currently critical for the accuracy of the prosthetic structures
3. The material from which the articulators are made—aluminum or carbon and fine details that make up the construction of these precision devices

With the device we offer, AROC, there is an opportunity for direct fixation of the phantom models, through a screw, without an intermediary.

The device AROC is designed to be able to withstand loads significantly exceeding the force used. The strength of the structure is guaranteed by the use of chromium-nickel (Cr-Ni) steel material and the solid thickness of the upper and lower plate.

In our in vitro study, the registration of contacts was performed on dry and smooth tooth surfaces and we did not encounter one of the disadvantages of AP—easy washing of the markings from saliva [7]. We found that after several repetitions (up to 3), the quality indicators are torn and begin to lose their marking ability. In the examination with the T-Scan system, it was found that in all repetitions the occlusal contacts that were registered were the same and the sensor did not rupture. This confirms the claim of the manufacturer that 15-25 recordings can be made with one sensor [29], and we reject the claim that the success of the T-Scan system has been negatively affected by the repeated use of the sensors [7].

Many studies have shown that the evaluation of contacts is subjective and allows for errors in occlusal corrections. Kerstein in his study proved that the visual assessment of the strength of the contacts registered with articulating paper in 87.7-88.2% of cases is wrong. Articulating foil and articulating paper cannot quantify the occlusal forces and the sequence of contact. When comparing the obtained markings between the quality indicators and the T-Scan system, it can be seen that the point markings reflect a higher occlusal pressure and the planar markings a lower force. These results confirm similar previous studies [20].

Examination of the occlusal films revealed that the third molars experienced a very high occlusal tension. These results confirm the phenomenon of domination of contacts in CO formulated by Filtchev [37] and later confirmed by Kalachev by the T-Scan system [38]. It reads as follows: “The maximum clenching forces of masticatory muscles form the strongest occlusal contacts on the most posterior teeth in 90% of cases at maximum pressure in CO of natural teeth.”

The benefits of using T-Scan in clinical practice have been proven for 37 years [29, 31]. Through the software of the system, we can determine the sequence of contacts in real time and the applied force on each tooth. Based on quantitative indicators that give much better and more accurate information, subjectivity is eliminated in the interpretation of contacts, which reduces the risk of errors. To visualize the location of the registered occlusal contacts with the system, it is intraoral to use a quality indicator, and we recommend the use of AF. The proposal is based on the statistically significant difference found between the number of contacts with AP and AF. Once again, our study confirms that the T-Scan system is a useful and necessary tool in the study and correction of occlusion.

## 6. Conclusion

Based on findings from the current in vitro simulation, we can conclude that the size and intensity of the markings obtained with articulating foil and articulating paper are not reliable criteria for assessing the strength of the contacts, as the interpretation is subjective. The T-Scan system has been proving for years that it is a useful and necessary tool in the study and correction of occlusion, as it is based on quantitative indicators, but it cannot be used to locate occlusal contacts intraorally. The type of occlusal indicator and the conditions of the oral environment in a clinical study have an impact on the registration of contacts. Therefore, we propose as a method of choice to achieve a balanced occlusion in clinical practice to combine the use of one conventional and one quantitative method.

## Figures and Tables

**Table 1:** Registered contacts according to the type of the occlusal indicator.

Occlusal indicators		Mean	Std. deviation	Std. error	95% confidence interval for mean		Minimum	Maximum
					Lower bound	Upper bound		
Tooth number								
18	12	4.50	0.527	0.167	4.12	4.88	4	5
	100	2.70	0.675	0.213	2.22	3.18	2	4
17	12	5.10	0.738	0.233	4.57	5.63	4	6
	100	4.60	0.699	0.221	4.10	5.10	4	6
16	12	4.70	0.483	0.153	4.35	5.05	4	5
	100	4.30	0.675	0.213	3.82	4.78	4	6
15	12	2.40	0.699	0.221	1.90	2.90	1	3
	100	1.90	0.568	0.180	1.49	2.31	1	3
14	12	2.40	0.699	0.221	1.90	2.90	1	3
	100	2.00	0.471	0.149	1.66	2.34	1	3
13	12	1.00	0.000	0.000	1.00	1.00	1	1
	100	1.00	0.000	0.000	1.00	1.00	1	1
12	12	0.90	0.316	0.100	0.67	1.13	0	1
	100	1.00	0.000	0.000	1.00	1.00	1	1
11	12	0.90	0.316	0.100	0.67	1.13	0	1
	100	0.10	0.316	0.100	-0.13	0.33	0	1
21	12	0.30	0.483	0.153	-0.05	0.65	0	1
	100	0.10	0.316	0.100	-0.13	0.33	0	1
22	12	1.00	0.000	0.000	1.00	1.00	1	1
	100	1.00	0.000	0.000	1.00	1.00	1	1
23	12	1.00	0.000	0.000	1.00	1.00	1	1
	100	1.10	0.316	0.100	0.87	1.33	1	2
24	12	2.50	0.527	0.167	2.12	2.88	2	3
	100	1.10	0.316	0.100	0.87	1.33	1	2
25	12	2.50	0.527	0.167	2.12	2.88	2	3
	100	1.40	0.516	0.163	1.03	1.77	1	2
26	12	4.50	0.527	0.167	4.12	4.88	4	5
	100	4.20	0.632	0.200	3.75	4.65	3	5
27	12	4.50	0.527	0.167	4.12	4.88	4	5
	100	4.20	0.422	0.133	3.90	4.50	4	5
28	12	5.20	0.789	0.249	4.64	5.76	4	6
	100	3.20	0.632	0.200	2.75	3.65	2	4
38	12	4.70	0.483	0.153	4.35	5.05	4	5
	100	3.60	0.699	0.221	3.10	4.10	2	4
37	12	4.00	0.471	0.149	3.66	4.34	3	5
	100	4.20	0.422	0.133	3.90	4.50	4	5
36	12	3.90	0.316	0.100	3.67	4.13	3	4
	100	4.56	0.527	0.176	4.15	4.96	4	5
35	12	2.40	0.699	0.221	1.90	2.90	1	3
	100	1.60	0.516	0.163	1.23	1.97	1	2
34	12	2.50	0.707	0.224	1.99	3.01	1	3
	100	1.60	0.516	0.163	1.23	1.97	1	2
33	12	1.00	0.000	0.000	1.00	1.00	1	1
	100	1.40	0.516	0.163	1.03	1.77	1	2
32	12	0.00	0.000	0.000	0.00	0.00	0	0
	100	0.00	0.000	0.000	0.00	0.00	0	0
31	12	0.00	0.000	0.000	0.00	0.00	0	0
	100	0.00	0.000	0.000	0.00	0.00	0	0
41	12	0.00	0.000	0.000	0.00	0.00	0	0
	100	0.00	0.000	0.000	0.00	0.00	0	0
42	12	0.00	0.000	0.000	0.00	0.00	0	0
	100	0.10	0.316	0.100	-0.13	0.33	0	1
43	12	1.00	0.000	0.000	1.00	1.00	1	1
	100	1.20	0.632	0.200	0.75	1.65	1	3
44	12	1.20	0.422	0.133	0.90	1.50	1	2
	100	2.10	0.568	0.180	1.69	2.51	1	3
45	12	1.70	0.483	0.153	1.35	2.05	1	2
	100	2.60	0.516	0.163	2.23	2.97	2	3
46	12	4.70	0.483	0.153	4.35	5.05	4	5
	100	4.20	0.632	0.200	3.75	4.65	4	6
47	12	4.60	0.516	0.163	4.23	4.97	4	5
	100	4.30	0.675	0.213	3.82	4.78	3	5
48	12	4.40	0.699	0.221	3.90	4.90	3	5
	100	2.60	0.966	0.306	1.91	3.29	2	5

**Table 2:** Comparison of the registered contacts.

Tooth number	Mean difference (*I* − *J*)	Std. error	Sig.	95% confidence interval	
				Lower bound	Upper bound
18	1.800^∗^	0.437	0.001	0.56	3.04
17	0.500^∗^	0.289	0.427	-0.32	1.32
16	0.400^∗^	0.192	0.245	-0.15	0.95
15	0.500	0.244	0.260	-0.19	1.19
14	0.400	0.224	0.395	-0.24	1.04
13	0.000	0.089	1.000	-0.25	0.25
12	-0.100^∗^	0.089	0.796	-0.35	0.15
11	-0.100^∗^	0.089	0.796	-0.35	0.15
21	0.200	0.167	0.754	-0.28	0.68
22	0.000	0.141	1.000	-0.40	0.40
23	-0.100	0.089	0.796	-0.35	0.15
24	1.400^∗^	0.192	0.000	0.85	1.95
25	1.100	0.196	0.000	0.54	1.66
26	0.300	0.288	0.835	-0.52	1.12
27	0.300	0.240	0.724	-0.38	0.98
28	2.000^∗^	0.288	0.000	1.18	2.82
38	1.100	0.268	0.002	0.34	1.86
37	-0.200^∗^	0.200	0.854	-0.77	0.37
36	-0.656	0.286	0.166	-1.47	0.16
35	0.800	0.280	0.048	0.01	1.59
34	0.900	0.243	0.005	0.21	1.59
33	-0.400^∗^	0.133	0.034	-0.78	-0.02
32	0.000	0.105	1.000	-0.30	0.30
31	0.000	0.123	1.000	-0.35	0.35
41	0.000	0.063	1.000	-0.18	0.18
42	-0.100^∗^	0.063	0.517	-0.28	0.08
43	-0.200^∗^	0.171	0.769	-0.69	0.29
44	-0.900^∗^	0.196	0.000	-1.46	-0.34
45	-0.900^∗^	0.291	0.027	-1.73	-0.07
46	0.500^∗^	0.276	0.379	-0.28	1.28
47	0.300	0.288	0.834	-0.52	1.12
48	1.800	0.343	0.000	0.82	2.78

## Data Availability

Data are available on request.
